# The Role of Prenatal Neurosonography in Identification of Tubulinopathy—Narrative Review

**DOI:** 10.3390/life16030501

**Published:** 2026-03-19

**Authors:** Krzysztof Berbeka, Katarzyna Stefańska, Aleksy Świetlicki, Dagmara Filipecka-Tyczka, Magda Rybak-Krzyszkowska, Miriam Illa

**Affiliations:** 1 First Department of Obstetrics and Gynecology, Centre of Postgraduate Medical Education, 01-004 Warsaw, Poland; berbekakrzysztof@gmail.com (K.B.); d.filipecka@gmail.com (D.F.-T.); 2 Department of Gynecology and Obstetrics, Medical University of Gdansk, 80-210 Gdansk, Poland; 3 Department of Reproduction, Poznan University of Medical Sciences, 60-535 Poznan, Poland; aleksy.swietlicki@gmail.com; 4 Doctoral School, Poznan University of Medical Sciences, 61-701 Poznan, Poland; 5 Department of Obstetrics and Perinatology, University Hospital, 30-688 Krakow, Poland; magda@hi-gen.pl; 6 Hi-Gen Centrum Medyczne, 30-552 Krakow, Poland; 7 BCNatal, Fetal Medicine Research Center (Hospital Clinic and Hospital Sant Joan de Déu), Universitat de Barcelona, 08950 Barcelona, Spain; miriam.illa@sjd.es; 8 Institut de Recerca Sant Joan de Déu (IRSJD), Fundació Sant Joan de Déu, 08950 Barcelona, Spain; 9 Spanish Network in Maternal, Neonatal, Child and Developmental Health Research (RICORS-SAMID, RD24/0013/0004), Instituto de Salud Carlos III, 28040 Madrid, Spain

**Keywords:** tubulinopathies, neurosonography, fetal brain malformations, malformations of cortical development

## Abstract

Tubulinopathies are severe, non-progressive neurodevelopmental disorders caused by mutations in tubulin genes, leading to profound intellectual disability, drug-resistant epilepsy, motor impairment, and lifelong dependence on care. While historically diagnosed postnatally, advances in prenatal neurosonography now allow for the suspicion of this pathology in utero during routine second and early third-trimester anomaly scans. This narrative review synthesizes key findings from the literature published between 2019 and 2025 regarding prenatal ultrasonographic signs of tubulinopathies. Recognition of specific red-flag patterns should prompt dedicated neurosonography and targeted genetic testing. Early pregnancy detection is crucial for parental counseling and evidence-based decisions regarding pregnancy continuation.

## 1. Introduction

Tubulinopathies are group of disorders caused by mutations in genes encoding tubulin isotypes, which disrupt neuronal migration. Tubulins assemble into microtubules—structures essential for cell division, axon guidance, and proper neuronal movement during brain development. These mutations weaken microtubule stability, leading to complex congenital brain malformations [1,2]. To date, pathogenic variants in seven tubulin genes have been implicated in these conditions [2,3,4].

Pathomorphological manifestations of the disease include basal ganglia dysmorphism with a fused striatum (pathognomonic in approximately 75% of cases), dysgenesis of the internal capsule (most commonly involving the anterior limb), and a broad spectrum of cortical anomalies. Associated features include ventriculomegaly, a small or absent corpus callosum, hypoplasia of the pons, dysgyria, and cerebellar hypoplasia or dysplasia [1,5]. Cortical patterns are heterogenous and may include microlissencephaly, agyria–pachygyria, central pachygyria, polymicrogyria-like dysplasia and simplified gyral patterns. Axonal pathfinding defects contribute to white matter and cranial nerve abnormalities. Ventriculomegaly, particularly when associated with cerebellar abnormalities and additional neurosonographic findings, may raise suspicion of a tubulinopathy.

Affected children typically present with severe neurological impairment in infancy including profound intellectual disability (often non-verbal), drug-resistant epilepsy (such as infantile spasms and focal seizures), spastic tetraparesis or diparesis with axial hypotonia, absent speech, swallowing difficulties requiring gastrostomy, and sensorimotor polyneuropathy. Additional features may include postnatal microcephaly, strabismus, and cranial nerve palsies. The condition is non-progressive but devastating; most patients require lifelong institutional care and have reduced life expectancy due to respiratory complications [6]. Nowadays, no curative therapy exists and management remains purely symptomatic.

Given this uniformly poor prognosis, prenatal diagnosis is essential. It allows parents to receive accurate and compassionate counseling and to make fully informed reproductive decisions, including termination of pregnancy where legally available. Ultrasound—routine, non-invasive, cost-effective, and universally accessible—plays a pivotal role as the first-line tool for raising suspicion.

Tubulinopathies are rare neurodevelopmental disorders, with an estimated prevalence of less than 1 in 1,000,000 for specific subtypes such as tubulinopathy-associated dysgyria (Orphanet: 467166) [7]. However, in selected cohorts of patients with complex malformations of cortical development (MCD), including lissencephaly, polymicrogyria, or microlissencephaly, pathogenic variants in tubulin genes account for a substantial proportion, ranging from approximately 10–40% depending on the series and selection criteria [8]. This highlights their relative importance among genetic causes of fetal brain malformations encountered in prenatal practice, particularly when ventriculomegaly, callosal anomalies, or cerebellar hypoplasia are identified on routine ultrasound, warranting targeted genetic evaluation.

Ultrasonographic signs reported in the recent literature (2019–2025) were collated to provide gynecologists and obstetric sonographers with a practical checklist. When these signs are identified on an anomaly scan, immediate referral for dedicated neurosonography and molecular testing (tubulin panel or exome sequencing) is warranted. This narrative review synthesizes the relevant literature on prenatal neurosonographic signs of tubulinopathies, identified through targeted searches in PubMed/MEDLINE and Google Scholar (keywords: tubulinopathy, prenatal ultrasound, fetal MRI, *TUBA1A*, *TUBB3*, etc.) for publications from 2019 to 2025. Articles were chosen for their focus on fetal imaging patterns and clinical implications in obstetric practice. The aim is to highlight that suggestive ultrasound findings, when recognized, can raise suspicion of tubulinopathy and should lead to dedicated neurosonography, fetal MRI, and targeted genetic testing for confirmation of the diagnosis.

## 2. Pathophysiology

Tubulinopathies arise from pathogenic variants in genes encoding different isotypes of tubulin, proteins that polymerize to form microtubules—dynamic cytoskeletal structures essential for multiple cellular processes during brain development. Microtubules serve as tracks for intracellular transport, facilitate mitotic spindle formation, enable axon guidance, and support neuronal migration from the ventricular zone to the cortical plate. They also contribute to cortical organization, maintenance of pial basement membrane integrity, and growth cone dynamics in axonal leading processes.

Seven main tubulin genes are implicated: α-tubulin (*TUBA1A, TUBA8*), β-tubulin (*TUBB2A, TUBB2B, TUBB3, TUBB/TUBB5*), and γ-tubulin (TUBG1) [2,3,4]. Mutations disrupt microtubule kinetics by altering tubulin heterodimer folding, GTP binding or hydrolysis, or incorporation into protofilaments, leading to unstable or dysfunctional microtubules. This impairment affects key neurodevelopmental processes, including progenitor cell proliferation in germinal matrices, radial glial scaffold function, neuronal motility, and post-migratory differentiation [8,9,10].

The resulting malformations reflect the temporal and spatial expression patterns of specific tubulin isotypes. For instance, *TUBA1A* mutations predominantly affect early neuronal migration, resulting in severe lissencephaly or microlissencephaly with thin parenchyma, an agyric cortex, and enlarged ventricles [11,12]. *TUBB2B* variants are frequently linked to polymicrogyria-like cortical dysplasia, with asymmetric perisylvian involvement and potential schizencephaly [13]. *TUBB3* mutations commonly cause dysgyria, basal ganglia dysmorphism, and brainstem or vermian hypoplasia, sometimes with congenital fibrosis of extraocular muscles (CFEOM) due to disrupted axon guidance [14]. Most variants arise de novo (>95%), though some (e.g., in *TUBB3* or *TUBB2B*) may be inherited from mildly affected or mosaic parents [1]. Extracerebral manifestations, such as ocular anomalies (strabismus, ptosis), sensorimotor polyneuropathy, pseudobulbar palsy with facial diplegia, and in rarer cases, more extensive syndromic features including arthrogryposis multiplex congenita or fetal akinesia deformation sequence, have been described. While many of these are considered secondary to central nervous system involvement (e.g., due to disrupted axonal guidance or motor neuron function), emerging evidence suggests that some extracerebral features, particularly severe neuromuscular involvement leading to fetal akinesia or arthrogryposis, may represent primary components of the tubulinopathy spectrum rather than purely secondary phenomena [15,16,17]. In prenatal settings, these disruptions may be detectable as early as the second trimester by neurosonography or fetal magnetic resonance imaging (MRI), particularly in severe forms with microcephaly, absent gyration, and pontocerebellar hypoplasia, facilitating early genetic testing and counseling [5,18].

## 3. Prenatal Neurosonographic Signs

Prenatal imaging findings in tubulinopathies represent a broad continuum that reflects the timing and extent of microtubule dysfunction during critical stages of brain development. Early and severe disruption, primarily affecting progenitor cell proliferation and initial neuronal migration, results in a “severe” prenatal imaging phenotype characterized by gross architectural abnormalities, such as microlissencephaly, extreme microcephaly, agyric cortex, severe ventriculomegaly, and severe pontocerebellar hypoplasia. These features are usually readily detectable on a mid-gestation ultrasound or fetal MRI, often as early as 21–24 weeks GA. In contrast, later or partial impairment—mainly involving cortical organization, late migratory processes, and axonal guidance—gives rise to a “mild” prenatal imaging phenotype, defined by more subtle, frequently asymmetric, and sometimes overlooked abnormalities, such as mild ventriculomegaly, callosal dysgenesis, dysmorphic basal ganglia, distorted midline structures, delayed Sylvian fissure operculization, and dysgyria. Importantly, this imaging-based distinction primarily reflects differences in prenatal detectability rather than necessarily predicting postnatal clinical severity: both severe and mild imaging phenotypes are most often associated with significant neurodevelopmental impairment, drug-resistant epilepsy, and poor long-term neurological prognosis.

The diagnostic challenge predominantly lies with the mild forms of tubulinopathy, which constitute the focus of the recent literature and the majority of prenatally suspected cases in expert centers. These require a high index of suspicion, dedicated neurosonography, and frequently complementary fetal MRI to identify characteristic patterns.

Prenatal detection of tubulinopathies primarily relies on dedicated neurosonography, typically conducted in the late second or third trimester, often supplemented by fetal MRI for confirmation and improved anatomical detail. Initial suspicion frequently arises from routine ultrasound screening that identifies anomalies such as ventriculomegaly, callosal dysgenesis, or abnormal posterior fossa structures. In a multicenter cohort study by Hagege et al. including 34 fetuses with mild tubulinopathy, the mean gestational age at initial screening was 24.2 weeks of gestational age (GA) (range 17–33 weeks GA), with referrals mainly due to callosal anomalies (56%) or ventricular abnormalities (53%) [18]. Dedicated neurosonography was performed at a mean of 28.3 weeks GA (range 23–34 weeks GA), and MRI at 30.2 weeks GA (range 24–35 weeks GA). This timing allows for better visualization of subtle cortical and subcortical features as brain development progresses, although recent case reports suggest detectability as early as 21 weeks GA [12,19].

### 3.1. Common Findings

Common prenatal neurosonographic signs in mild tubulinopathy include a combination of ventricular, midline, and cortical abnormalities, often asymmetric and subtle. In the aforementioned cohort, ventriculomegaly was observed in 19/33 cases on ultrasound (mean atrial width 11 mm, range 10–14.9 mm) and 23/30 on MRI (mean 11.9 mm, range 10–19 mm), typically mild and unilateral. Cerebellar hypoplasia was noted on MRI, with reduced vermian height (≤5th centile in 14/29) and transverse cerebellar diameter within the normal limits in most cases. On ultrasound, vermian height was normal in most cases. Callosal anomalies, including dysgenesis, shortening, or partial agenesis, were detected in 19/33 on ultrasound and 12/30 on MRI. Absence or asymmetry of the olfactory sulci was seen in 11/17 on ultrasound and 9/18 on MRI, often evaluable only after 28–30 weeks. Basal ganglia dysmorphism, appearing as enlarged, asymmetric, hyperechoic structures below the frontal horns, was identified in 18/30 on ultrasound but less frequently (6/28) on MRI. MRI frequently reveals subtle abnormalities missed on ultrasound, such as dysgyria (abnormal sulcal orientation with irregular depths) in all 29/29 cases examined, ventricular distortion (27/29), and brainstem anomalies like reduced anteroposterior pons diameter (≤5th centile in 17/29) or abnormal bulge (20/29) [20].

Extracerebral findings are rare but may include a dysplastic pelvic kidney or single umbilical artery, as noted in isolated cases. Recent studies emphasize the utility of Sylvian fissure biometry for detecting cortical abnormalities, with new reference charts aiding in identifying delayed operculization or asymmetry indicative of tubulinopathies [21].

It is important to acknowledge the limitations of prenatal ultrasound in detecting tubulinopathies. Mild forms often present with subtle, asymmetric, and operator-dependent findings that may be overlooked on routine scans, particularly before 28 weeks of gestation. Interobserver variability is significant, and dedicated expert neurosonography is essential for reliable identification of characteristic patterns. Fetal MRI frequently provides superior detail for subtle abnormalities (e.g., dysgyria, basal ganglia dysmorphism, or brainstem changes) and should complement ultrasound in suspected cases. Definitive diagnosis requires molecular genetic confirmation through targeted sequencing or exome analysis.

### 3.2. Spectrum of Patterns

Prenatal imaging reveals a spectrum from severe to mild forms, with mild tubulinopathy characterized by subtle, often asymmetric anomalies detectable from mid-gestation [22].

• Severe Form: Typically linked to *TUBA1A* mutations; this presents early (around 23 weeks GA) with enlarged germinal matrices, microlissencephaly (extreme microcephaly with absent gyration), kinked or thin brainstem, parenchymal thinning, agyric cortex, markedly enlarged ventricles, severe vermian hypoplasia, and pontocerebellar atrophy [23]. These features are more readily apparent on early scans and often lead to poor prognosis.
• Mild Form: More common in this review’s focus, featuring non-specific but suggestive signs like asymmetric brainstem (minor criterion on MRI in 20/29), corpus callosum (CC) dysgenesis (e.g., thin or short CC), lack of Sylvian fissure operculization (asymmetric in 18/23 on ultrasound), and distortion of the anterior interhemispheric fissure with frontal lobe impaction or interdigitation [21,23]. Basal ganglia often show fusion of lenticular and caudate nuclei without a visible anterior limb of internal capsule, appearing hooked or rounded on imaging.

In the cohort of *n* = 34 fetuses with mild tubulinopathy, major ultrasound criteria (prevalence ≥ 70%) included midline distortion (22/29, often anterior interhemispheric fissure deviation), ventricular asymmetry (26/30, difference ≥ 2 mm), dysmorphic frontal horns (26/30, lateral > medial portion with hooked appearance), dilated frontal horns (27/29, mostly unilateral), and abnormal sulcation (23/30, suggestive of dysgyria with irregular, obliquely oriented sulci). Minor ultrasound criteria (50–70%) included cavum septi pellucidi distortion (15/26, deviated or displaced), callosal anomalies (19/33), absent/asymmetric olfactory sulci (11/17), ventriculomegaly (19/33), and basal ganglia dysmorphism (18/30) [18]. Subependymal pseudocysts were occasional (5/30 on ultrasound, 3/29 on MRI), appearing as anechoic/hypoechoic cysts without layering.

Major MRI criteria mirrored these but with higher sensitivity: midline distortion (27/30), cavum septi pellucidi distortion (23/29), ventricular asymmetry/dilatation/distortion (29/30 asymmetry, 23/30 ventriculomegaly, 27/29 distortion with bowing and lateral concavity), dysmorphic/dilated frontal horns (23/29 dysmorphic, 27/29 dilated), and abnormal sulcation (29/29, predominantly dysgyria). Minor MRI criteria included absent/asymmetric olfactory sulci (9/18), reduced pons anteroposterior diameter (17/29), abnormal pons bulge (20/29, flattened anteriorly), and brainstem asymmetry (20/29, affecting pons or peduncles) [18].

Prenatal ultrasound criteria for mild tubulinopathy (based on a multicenter cohort of 34 fetuses of Hagege R et al.) are shown in Table 1 [18].

### 3.3. Gene-Specific Associations

Phenotypic variations partially correlate with the affected genes, though overlaps are common [2,24]:

• *TUBA1A* (17.6% of cases): Often associated with severe lissencephaly or microlissencephaly, marked ventriculomegaly, callosal dysgenesis, and vermian hypoplasia; in milder forms, early presentation with hooked frontal horns and dysgyria. Novel variants such as *TUBA1A* c.799T>C and c.1204C>T (p.Arg402Cys) expand the phenotypic spectrum, enabling earlier prenatal detection (as early as 21 weeks) and highlighting associations with epileptic spasms [24,25,26,27,28].
• *TUBB2B* (14.7% of cases): Linked to polymicrogyria-like cortical dysplasia, asymmetric perisylvian involvement, schizencephaly, asymmetric brainstem or cerebellum, and ventriculomegaly; subtle sulcal anomalies prominent [26].
• *TUBB3* (44.1% of cases): Commonly causes dysgyria, basal ganglia dysmorphism, brainstem/vermian hypoplasia, and milder prenatal phenotypes; ultrasound frequently identifies callosal and sulcal anomalies, sometimes with congenital fibrosis of extraocular muscles postnatally [29].
• *TUBB* (23.5%): Variable presentations, often mild with simplified gyral patterns, absence of microcephaly, and more frequent inheritance; subtle ventricular and midline distortions [26].
• Other (e.g., *TUBGCP2*, *TUBG1*): Rare, with features like thick corpus callosum or cerebellar neurodegeneration [30].

Inheritance was seen in 18 out of 34 cases (53%) in the group of mild tubulinopathies, usually passed on from parents with mild symptoms or mosaic changes [14]. In severe forms, mutations are almost always de novo (>95%), because these changes are much more harmful and parents with them rarely have children. The higher number of inherited cases in milder forms comes from two main reasons: some mutations are less harmful (so they can be passed to the child) and prenatal studies often find these milder, survivable changes [18,31].

Exome sequencing with research reanalysis has high utility in confirming diagnoses, especially in unresolved brain malformations [31,32].

Table 2 presents a summary of gene-specific prenatal ultrasound signs.

## 4. Differential Diagnosis

Tubulinopathies may mimic other cortical malformations (e.g., Walker–Warburg syndrome, mTORopathies). They can be distinguished by the lack of extracerebral signs and specific combinations, such as ventriculomegaly with cerebellar hypoplasia in the absence of severe ventriculomegaly. MRI helps differentiate dysgyria from true polymicrogyria [33]. Dysgyria in tubulinopathies typically appears on fetal MRI or dedicated neurosonography as irregular, shallow, obliquely oriented sulci with variable depths and poor operculization, often asymmetric and without the classic ‘cobblestone’ or excessively thick cortex seen in polymicrogyria. In contrast, true polymicrogyria shows multiple small, tightly packed gyri with an irregular cortical–white matter junction, frequently bilateral and perisylvian, and may be associated with schizencephaly in some cases. Distinguishing these patterns requires high-resolution imaging, preferably fetal MRI after 28 weeks, as ultrasound alone may not provide sufficient detail for reliable differentiation.

When ventriculomegaly is accompanied by severe cerebellar hypoplasia, pontine thinning, or eye/muscle abnormalities, dystroglycanopathies (e.g., Walker–Warburg syndrome) should be prioritized in the differential diagnosis and tested for early. Similarly, if cortical malformations are accompanied by tubers, subependymal nodules, or cardiac/renal findings, this should lead to genetic testing towards mTOR-related disorders (e.g., tuberous sclerosis). In the absence of these extracerebral or syndromic features, tubulin gene sequencing remains the most appropriate next step.

Recent reviews emphasize a comprehensive diagnostic work-up for malformations of cortical development, including genetic testing to rule out lissencephaly syndromes, α-dystroglycanopathies, or RELN-related disorders [29]. Parental or trio MRI can further refine diagnosis by identifying mild parental phenotypes, improving counseling accuracy in inherited cases [34]. Optic nerve hypoplasia may overlap with septo-optic dysplasia, but tubulin gene sequencing helps to differentiate between these entities [26].

Differentiation of symptoms from other pathologies is presented in Table 3.

## 5. Implications for Prenatal Counseling

Detection enables exome sequencing for diagnostic confirmation. Outcomes range from severe impairment to milder forms, with epilepsy and motor delay risks. Inheritance is observed in approximately 53% of cases [18]. Counseling should address phenotypic variability, reproductive options, and potential for atypical seizures like infantile spasms [28]. Particular attention should be paid to potential intrafamilial differences in neurological and developmental outcomes, especially in inherited cases involving genes such as *TUBB3*. Pathogenic variants in *TUBB3* can exhibit striking intrafamilial variability, with carriers in the same family displaying a wide range of phenotypes—from normal cognition and mild motor impairment to more severe neurological deficits—highlighting challenges in accurate prognosis and genetic counseling [35]. Parental or trio MRI enhances counseling by revealing subclinical parental anomalies, aiding recurrence risk assessment and family planning [34]. New charts for Sylvian fissure biometry support earlier detection of cortical delays, informing clinical decision-making [21]. Exome reanalysis in unresolved cases can yield diagnoses in up to 20–30% [32].

Early ultrasound suspicion enables rapid genetic confirmation and truly informed choice. Parents should be informed about the typically severe neurodevelopmental prognosis in most cases of tubulinopathy, which often includes profound intellectual disability, drug-resistant epilepsy, significant motor impairment, and a high likelihood of lifelong support needs. However, the exact outcome can vary, depending on the specific genetic variant, degree of brain malformation, and individual factors. Given the phenotypic variability (including milder presentations in some inherited cases), counseling should emphasize the range of possible outcomes and the importance of a multidisciplinary approach (geneticists, fetal medicine specialists, psychologists) to support parents throughout the process. Counseling must remain empathetic, individualized, non-directive, and sensitive to legal, cultural, and personal contexts, allowing parents to make fully informed decisions based on their values and circumstances.

**Figure 1:**
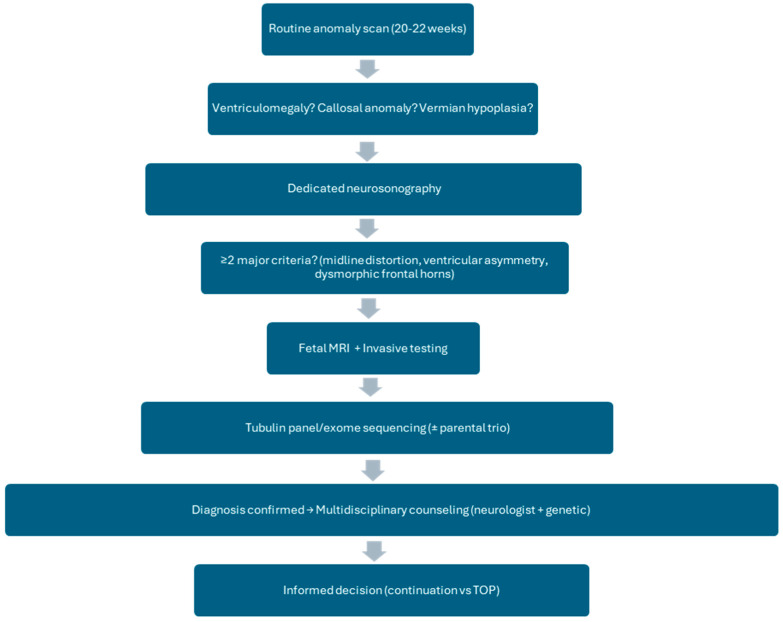
Diagnostic algorithm for suspected tubulinopathy. Flowchart illustrating the recommended diagnostic pathway starting from routine anomaly scan findings (e.g., ventriculomegaly, callosal anomaly, midline distortion) leading to dedicated neurosonography, fetal MRI, and targeted genetic testing. Created based on current clinical practice and literature [18,21].

Figure 1 presents the diagnostic algorithm for suspected tubulinopathy.

**Figure 2:**
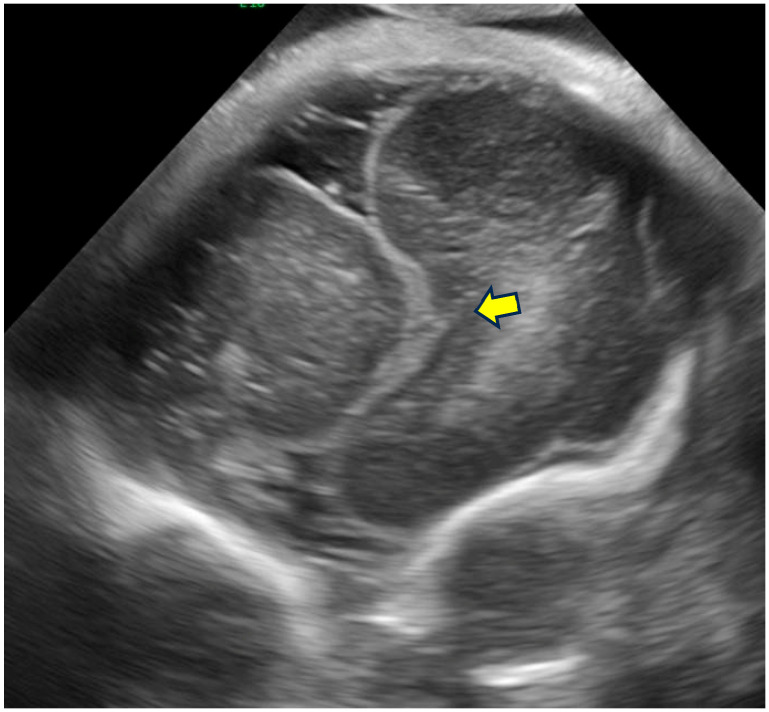
Coronal section through the frontal lobes showing deviation of the anterior interhemispheric fissure (arrow) and hemimegalencephaly. Representative image illustrating typical midline deviation in a suspected tubulinopathy case at approximately 28 weeks GA. Genetic confirmation was not available for this particular image.

**Figure 3:**
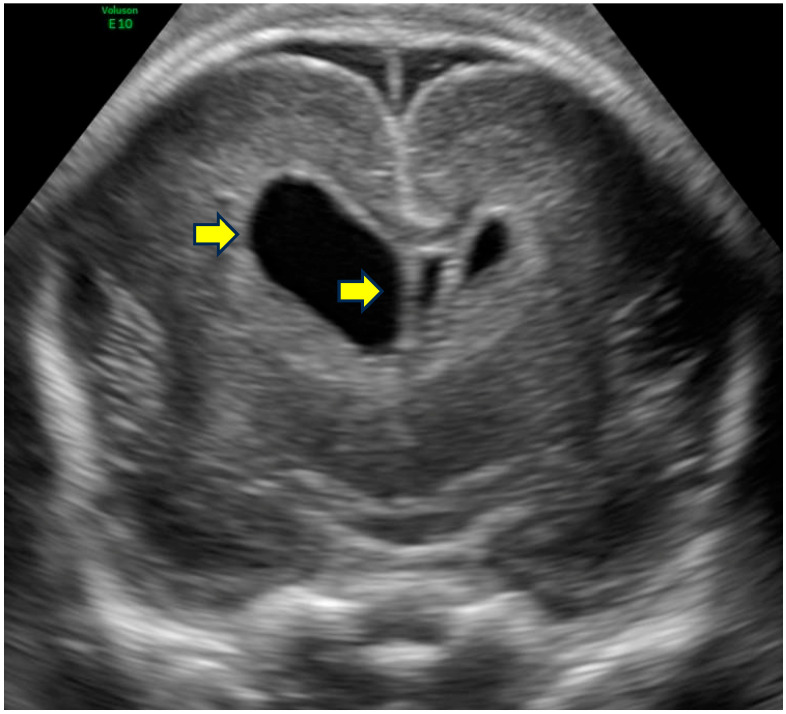
Coronal section showing asymmetry of the anterior horns and cavum septum pellucidum displacement. Fetal neurosonography at 22 weeks GA in a case with confirmed *TUBB3* variant. Arrows highlight dysmorphic and dilated frontal horns with displacement of the cavum septi pellucidi.

**Figure 4:**
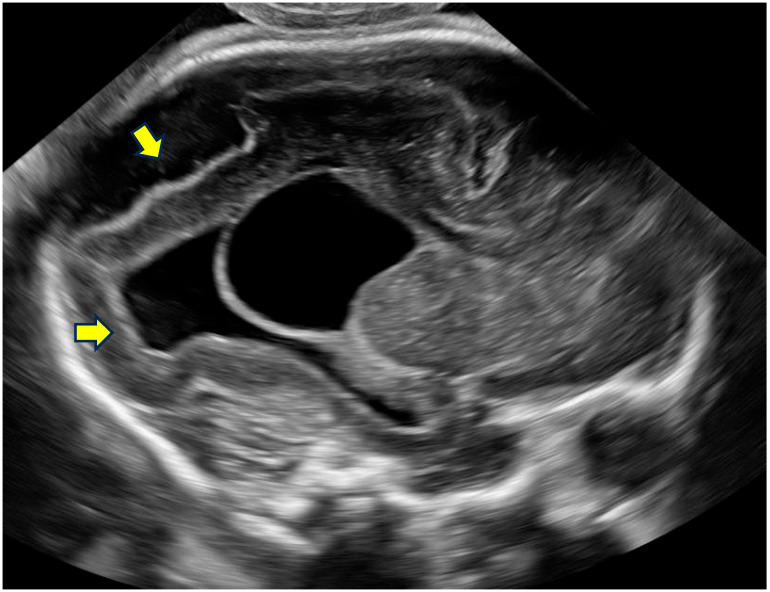
Parasagittal section showing severe ventriculomegaly, abnormal gyral pattern (dysgyria), and a choroid plexus cyst. Image from a genetically confirmed tubulinopathy at 32 weeks GA. Arrows point to irregular sulcation suggestive of dysgyria and marked ventriculomegaly.

**Figure 5:**
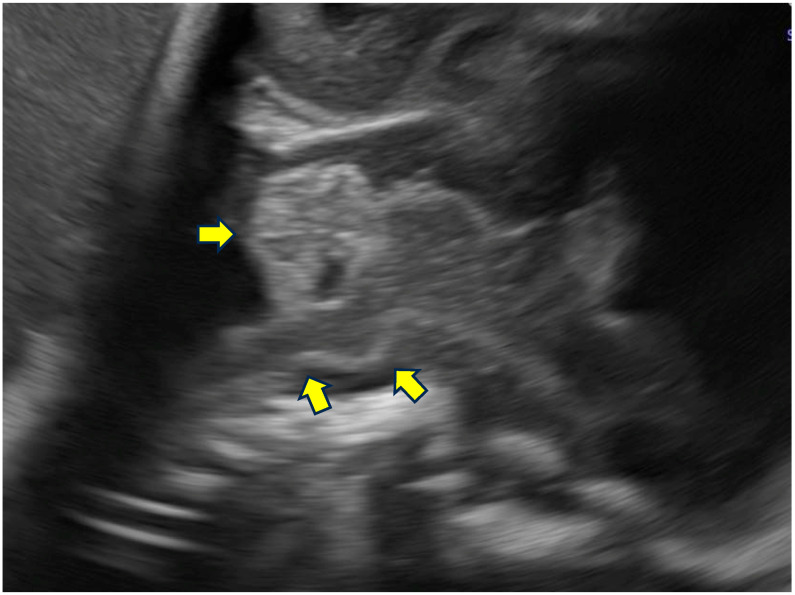
Sagittal section demonstrating kinking of the brainstem and a hypoplastic pons and cerebellar vermis. Representative sagittal view at 32 weeks GA in a severe tubulinopathy phenotype (*TUBA1A*-related). Arrows indicate brainstem kinking, thin pons, and vermian hypoplasia.

Representative examples of these characteristic neurosonographic findings are il-lustrated in Figure 2,Figure 3,Figure 4,Figure 5. Figure 2 shows a coronal view at approximately 28 weeks of gestation with clear deviation of the anterior interhemispheric fissure and frontal lobe interdigitation, highlighting midline distortion as a major criterion. Figure 3 demon-strates asymmetry of the frontal horns and displacement of the cavum septi pellucidi in a case at 22 weeks GA. Figure 4 depicts severe ventriculomegaly combined with ir-regular sulcation suggestive of dysgyria in a parasagittal section at 32 weeks, while Figure 5 illustrates brainstem kinking, thin pons, and vermian hypoplasia in a sagittal view at 32 weeks, typical of more severe phenotypes. These images, drawn from sus-pected or confirmed tubulinopathy cases, emphasize the value of targeted neuroso-nography in identifying subtle and asymmetric abnormalities that may be overlooked on routine scans.

## 6. Conclusions

Tubulinopathies represent one of the most severe prenatally detectable neurodevelopmental disorders. Routine obstetric screening ultrasound anomaly scans can raise suspicion in many cases, particularly when specific red-flag patterns (such as ventriculomegaly, callosal anomalies, or midline distortion) are present, although detection of mild forms remains challenging and often requires dedicated neurosonography. By recognizing the practical red flags outlined above, gynecologists can initiate a diagnostic cascade leading to molecular confirmation within days. This empowers parents with accurate prognostic information while reproductive options remain available. Future research should focus on strengthening genotype–phenotype correlations using larger, multicenter cohorts and the integration of cutting-edge imaging modalities—such as 3D/4D high-resolution neurosonography, long-read sequencing, and functional fetal MRI—which will enable more precise clinical prognostication and tailored prenatal counseling. While routine ultrasound plays a key role in initial suspicion, the diagnostic process is highly dependent on operator expertise and gestational age, and confirmation typically requires multidisciplinary input including fetal MRI and genetic testing.

## Figures and Tables

**Table 1:** Prenatal ultrasound criteria for mild tubulinopathy adapted from Hagege R et al. (based on multicenter cohort of 34 fetuses) ^1^.

Category	Ultrasound Sign	Prevalence	Description/What to Look for on An Anomaly Scan	Red Flag If Combined with …
**Major criteria** (≥70%—strongly suspect if ≥2 present)	Midline distortion	22/29 (76%)	Deviated anterior interhemispheric fissure (frontal lobes “pushed” or interdigitated)	Ventriculomegaly or callosal anomaly
	Ventricular asymmetry	26/30 (87%)	Difference ≥2 mm between lateral ventricles	Dysmorphic frontal horns
	Dysmorphic frontal horns	26/30 (87%)	Lateral > medial portion; “hooked” or rounded appearance	Basal ganglia changes
	Dilated frontal horns	27/29 (93%)	Unilateral dilatation (usually mild)	Abnormal sulcation
	Abnormal sulcation (dysgyria)	23/30 (77%)	Irregular, oblique, shallow sulci; lack of normal operculization	Cerebellar hypoplasia
**Minor criteria** (50–70%—evaluate if major signs present)	Cavum septi pellucidi distortion	15/26 (58%)	Deviated, displaced, or abnormally shaped	Midline distortion
	Corpus callosum anomalies	19/33 (58%)	Short, thin, partial agenesis, or dysgenesis	Ventriculomegaly
	Absent/asymmetric olfactory sulci	11/17 (65%)	Visible only >28 weeks; asymmetry or absence	Frontal horn changes
	Mild ventriculomegaly	19/33 (58%)	Atrial width 10–14.9 mm (usually unilateral)	Vermian hypoplasia
	Basal ganglia dysmorphism	18/30 (60%)	Enlarged, hyperechoic, fused caudate-putamen (no visible anterior internal capsule)	Hooked frontal horns

^1^ Adapted from Hagege R et al. 2023 [18]. Practical tip—High-suspicion triad (detectable on routine scan): Mild ventriculomegaly + callosal anomaly + vermian hypoplasia → Immediate referral for dedicated neurosonography.

**Table 2:** Gene-specific prenatal ultrasound signs.

Gene	Prevalence in Cohorts	Key Prenatal USG Signs (Major)	Earliest Detection	Postnatal Seizures/Spasms	Inheritance	References
***TUBA1A***	17–25%	Hooked frontal horns, severe vermian hypoplasia, microlissencephaly	21 weeks	Yes (infantile spasms common)	De novo 95%	Bornstein 2025 [25], Ren 2024 [27], Pavone 2023 [24]
***TUBB2B***	15%	Asymmetric polymicrogyria-like, schizencephaly, perisylvian dysplasia	24–28 weeks	Yes (focal)	Inherited ~30%	Hwang 2025 [26], Hagege 2023 [18]
***TUBB3***	44%	Dysgyria, basal ganglia fusion, brainstem asymmetry	23–30 weeks	Yes (drug-resistant)	Inherited ~50%	Blumkin 2020 [4], Ikegawa 2025 [23]
***TUBB***	23%	Simplified gyration, mild midline distortion	26–32 weeks	Variable	Inherited ~60%	Hagege 2023 [18]
**Others** (*TUBG1*, *TUBGCP2*)	<5%	Thick CC, cerebellar dysplasia	28+ weeks	Yes	Variable	Esen 2025 [30], Brock 2018 [12]

**Table 3:** Differential diagnosis—tubulinopathy vs. look-alikes.

Feature	Tubulinopathy	Lissencephaly (ARX/LIS1)	mTORopathy	Walker–Warburg
Ventriculomegaly	Mild, asymmetric	Severe, symmetric	Yes (severe)	Yes
Cortical pattern	Dysgyria (oblique sulci)	Agyria–pachygyria	Polymicrogyria	Cobblestone
Basal ganglia	Fused (pathognomonic)	Normal	Normal	Normal
Cerebellar vermis	Hypoplasia (no DWM)	Normal	Hypoplasia	DWM common
Extracerebral signs	Rare (optic hypoplasia)	No	Tuberous sclerosis	Eye, muscle
Genetic yield	>90% if ≥3 criteria	Variable	High	High

## Data Availability

No new data were created or analyzed in this study.

## References

[1] Bahi-Buisson N., Maillard C., Adam M.P., Feldman J., Mirzaa G.M., Pagon R.A., Wallace S.E., Amemiya A. (2016). Tubulinopathies Overview. 2016 Mar 24 [updated 2021 Sep 16]. GeneReviews^®^ [Internet].

[2] Romero D.M., Bahi-Buisson N., Francis F. (2018). Genetics and mechanisms leading to human cortical malformations. Semin. Cell Dev. Biol..

[3] Fallet-Bianco C., Laquerrière A., Poirier K., Razavi F., Guimiot F., Dias P., Loeuillet L., Lascelles K., Beldjord C., Carion N. (2014). Mutations in tubulin genes are frequent causes of various foetal malformations of cortical development including microlissencephaly. Acta Neuropathol. Commun..

[4] Blumkin L., Leibovitz Z., Krajden-Haratz K., Arad A., Yosovich K., Gindes L., Zerem A., Ben-Sira L., Lev D., Nissenkorn A. (2020). Autosomal dominant TUBB3-related syndrome: Fetal, radiologic, clinical and morphological features. Eur. J. Paediatr. Neurol..

[5] Brar B.K., Thompson M.G., Vora N.L., Gilmore K., Blakemore K., Miller K.A., Giordano J., Dufke A., Wong B., Stover S. (2022). Prenatal phenotyping of fetal tubulinopathies: A multicenter retrospective case series. Prenat. Diagn..

[6] Hung K.L., Lu J.F., Su D.J., Hsu S.J., Wang L.C. (2022). Tubulinopathy Presenting as Developmental and Epileptic Encephalopathy. Children.

[7] Schröter J., Döring J.H., Garbade S.F., Hoffmann G.F., Kölker S., Ries M., Syrbe S. (2021). Cross-sectional quantitative analysis of the natural history of TUBA1A and TUBB2B tubulinopathies. Genet. Med..

[8] Bahi-Buisson N., Poirier K., Fourniol F., Saillour Y., Valence S., Lebrun N., Hully M., Bianco C.F., Boddaert N., Elie C. (2014). The wide spectrum of tubulinopathies: What are the key features for the diagnosis?. Brain.

[9] Jaglin X.H., Chelly J. (2009). Tubulin-related cortical dysgeneses: Microtubule dysfunction underlying neuronal migration defects. Trends Genet..

[10] Cabet S., Putoux A., Buenerd A., Gueneau L., Reymond A., Thia E.W.H., Lai A.H.M., Schindewolf E.M., Sanlaville D., Lesca G. (2020). Prenatal cerebral imaging features of a new syndromic entity related to, K.IAA1109 pathogenic variants mimicking tubulinopathy. Prenat. Diagn..

[11] Cabet S., Karl K., Garel C., Delius M., Hartung J., Lesca G., Chaoui R., Guibaud L. (2021). Two different prenatal imaging cerebral patterns of tubulinopathy. Ultrasound Obstet. Gynecol..

[12] Brock S., Stouffs K., Scalais E., D’Hooghe M., Keymolen K., Guerrini R., Dobyns W.B., Di Donato N., Jansen A.C. (2018). Tubulinopathies continued: Refining the phenotypic spectrum associated with variants in *TUBG1*. Eur. J. Hum. Genet..

[13] Jaglin X.H., Poirier K., Saillour Y., Buhler E., Tian G., Bahi-Buisson N., Fallet-Bianco C., Phan-Dinh-Tuy F., Kong X.P., Bomont P. (2009). Mutations in the beta-tubulin gene TUBB2B result in asymmetrical polymicrogyria. Nat. Genet..

[14] Guduru M., Powers A., Love T., Beavers A. (2020). Second trimester fetal MRI features in a fetus with TUBB3 gene mutation. Radiol. Case Rep..

[15] Weber M., Jaber D., Encha-Razavi F., Julien E., Grevoul-Fesquet J., Steffann J., Melki J., Martinovic J. (2022). Broadening the phenotypic spectrum of TUBA1A tubulinopathy to syndromic arthrogryposis multiplex congenita. Am. J. Med. Genet. A.

[16] Li D., Shen K.M., Zackai E.H., Bhoj E.J. (2020). Clinical variability of TUBB-associated disorders: Diagnosis through reanalysis. Am. J. Med. Genet. A.

[17] Laquerriere A., Gonzales M., Saillour Y., Cavallin M., Joyē N., Quēlin C., Bidat L., Dommergues M., Plessis G., Encha-Razavi F. (2016). De novo TUBB2B mutation causes fetal akinesia deformation sequence with microlissencephaly: An unusual presentation of tubulinopathy. Eur. J. Med. Genet..

[18] Hagege R., Krajden Haratz K., Malinger G., Ben-Sira L., Leibovitz Z., Heron D., Burglen L., Birnbaum R., Valence S., Keren B. (2023). Spectrum of brain malformations in fetuses with mild tubulinopathy. Ultrasound Obstet. Gynecol..

[19] Ramirez D.A., Anninger W.V., Scoles D. (2025). Optic nerve hypoplasia and bilateral persistent fetal vasculature due to TUBA1A tubulinopathy. Retin Cases Brief Rep..

[20] Bekiesinska-Figatowska M., Romaniuk-Doroszewska A., Duczkowska A., Duczkowski M., Iwanowska B., Szkudlińska-Pawlak S. (2016). Fetal MRI versus postnatal imaging in the MR-compatible incubator. Radiol. Med..

[21] Peero E.K., Kugelman N., Gindes L., Shariv A., Lev D., Tamarkin M., Haddad L., Bakry H., Weizman B., Shapiro I. (2023). Diagnosis of fetal cortical abnormalities by new reference charts for assessment of sylvian fissure biometry. Prenat. Diagn..

[22] Sebire N.J., Miller S., Jacques T.S., Taylor A.M., Rennie J.M., Kendall G., Chitty L.S. (2013). Post-mortem apparent resolution of fetal ventriculomegaly: Evidence from magnetic resonance imaging. Prenat. Diagn..

[23] Ikegawa T., Osada K., Ikeda A., Tsuyusaki Y., Tsuji M., Iai M., Aida N., Kurosawa K., Matsumoto N., Goto T. (2025). Clinical characteristics and radiological features of tubulinopathy: A single-center retrospective study in Japan. Brain Dev..

[24] Pavone P., Striano P., Cacciaguerra G., Marino S.D., Parano E., Pappalardo X.G., Falsaperla R., Ruggieri M. (2023). Case report: Structural brain abnormalities in *TUBA1A*-tubulinopathies: A narrative review. Front. Pediatr..

[25] Bornstein E., Jobanputra V., Reiss S., Thomas-Wilson A., Baptiste C., Levy B., Malinger G. (2025). Prenatal Diagnosis of Tubulinopathy: Case Report of Neurosonographic Features and a Novel TUBA1A Variant. Fetal Diagn Ther..

[26] Hwang S., Bae H., Kim D., Yum M.S., Kim M.J., Yoon J.H., Kim J.H., Seo G.H., Kim G.H., Do H. (2025). Expanding genetic and clinical spectra of β-tubulinopathies: A Korean study. J. Hum. Genet..

[27] Ren S., Kong Y., Liu R., Li Q., Shen X., Kong Q.X. (2024). Lissencephaly caused by a de novo mutation in tubulin *TUBA1A*: A case report and literature review. Front. Pediatr..

[28] Ng A.C., Scantlebury M.H. (2025). TUBA1A-related tubulinopathy associated with the infantile epileptic spasms syndrome and atypical absence seizures. Epileptic Disord..

[29] Rijckmans E., Stouffs K., Jansen A.C. (2024). Diagnostic work-up in malformations of cortical development. Dev. Med. Child Neurol..

[30] Esen T.E., Ardiçli D., Keçelİ A.M., Çavdarli B. (2025). Thick Corpus Callosum: An Unusual Finding of TUBGCP2-Related Tubulinopathy. Am. J. Med. Genet. A.

[31] Zillhardt J.L., Poirier K., Broix L., Lebrun N., Elmorjani A., Martinovic J., Saillour Y., Muraca G., Nectoux J., Bessieres B. (2016). Mosaic parental germline mutations causing recurrent forms of malformations of cortical development. Eur. J. Hum. Genet..

[32] Kooshavar D., Amor D.J., Boggs K., Baker N., Barnett C., de Silva M.G., Edwards S., Fahey M.C., Marum J.E., Snell P. (2024). Diagnostic utility of exome sequencing followed by research reanalysis in human brain malformations. Brain Commun..

[33] Charan B.D., Gaikwad S.B., Dixit T. (2025). Characteristic Imaging Feature of Cortical Dysgenesis in Tubulinopathy. World Neurosurg..

[34] Rodó C., Maiz N., Vázquez É., Gómez-Andrés D., Valenzuela I., Abulí A., Carreras E. (2024). Parental or Trio Magnetic Resonance Imaging to Improve Prenatal Counseling in Brain Anomalies. Prenat. Diagn..

[35] Bouazzaoui A., Quélin C., Rozel C., Carré W., Dubourg C., Odent S., Rollier P. (2025). Expanding the TUBB3-Related Phenotypic Landscape: Fetal Diagnosis of Novel TUBB3 Variant Linked with Phenotypic Variability Within a Single Family. Prenat. Diagn..

